# Routine Online Psychological Therapy in an Insurance-Based Care Setting: Retrospective Service Evaluation of Real-World Outcomes

**DOI:** 10.2196/101074

**Published:** 2026-09-25

**Authors:** Tobias Opsahl, Mikkel Meinert, Emil Brødsgaard

**Affiliations:** 1 We.Care Health A/S Copenhagen Denmark

**Keywords:** online psychological therapy, video-based psychotherapy, insurance-based mental health services, real-world evidence, depression, anxiety, perceived stress

## Abstract

**Background:**

Depression, anxiety, and stress-related difficulties represent a major global health burden. Online psychological therapy has emerged as a promising approach to increasing access to care, yet evidence from routine, real-world clinical settings, particularly for services delivered without standardized treatment protocols, remains limited.

**Objective:**

This study aimed to describe symptom change and patient-reported satisfaction associated with routine online psychological therapy delivered within an insurance-based care setting.

**Methods:**

A retrospective observational service evaluation was conducted using deidentified routine care data. Clients aged 15 years and older who initiated online psychological therapy and completed baseline and end-of-treatment assessments on all 3 outcome measures were included. Symptoms of depression, anxiety, and perceived stress were measured using the 9-item Patient Health Questionnaire (PHQ-9), 7-item Generalized Anxiety Disorder (GAD-7), and 10-item Perceived Stress Scale (PSS-10), respectively. Changes in symptoms were examined using paired sample *t* tests, within-sample effect sizes (Cohen *d* and Cohen *d_z_* with 95% CIs), and reliable change indices. Robustness was assessed with therapist-clustered mixed models and attrition sensitivity analyses. A ≥50% reduction in symptom scores was reported as a descriptive response criterion. Patient satisfaction was assessed with single-item ratings at the end-of-treatment assessment.

**Results:**

A total of 1221 clients were included. Clients completed a mean of 4.7 (SD 1.67) therapy sessions over an average treatment duration of 62.7 (SD 40.4) days. Significant reductions with large within-sample effect sizes were observed for depression (Cohen *d*=1.25, 95% CI 1.17-1.32), anxiety (Cohen *d*=1.49, 95% CI 1.41-1.57), and perceived stress (Cohen *d*=1.46, 95% CI 1.38-1.54). A ≥50% symptom reduction was observed in 60.7% (741/1220) of clients for depression, 67.5% (824/1221) for anxiety, and 30.8% (376/1221) for perceived stress. At the end-of-treatment assessment, 80.7% (985/1221) scored below the clinical cutoff for depression (PHQ-9<10) and 79.2% (967/1221) for anxiety (GAD-7<8). Reliable improvement was observed in 56% (688/1221) to 65% (794/1221) of clients, with deterioration below 2% on all measures. Mean patient-reported satisfaction scores ranged from 8.55 (SD 1.71) to 8.96 (SD 1.45) on a 10-point scale, and mean recommendation likelihood was 8.61 (SD 1.88). Therapist-level intraclass correlation coefficients were ≤0.029; effect sizes were 1.27-1.50 under inverse-probability weighting and 0.41-0.50 under zero-change imputation for baseline-screened clients without end-of-treatment assessments.

**Conclusions:**

Routine online psychological therapy delivered within an insurance-based care setting was associated with substantial pre-post symptom reductions, high proportions of clients meeting a descriptive ≥50% reduction criterion and showing reliable improvement, and high patient-reported satisfaction. Because the evaluation used an uncontrolled pre-post design restricted to clients with paired assessments, observed changes cannot be attributed causally to treatment. High satisfaction among responding clients is consistent with acceptability of the model to those clients; feasibility was not formally assessed, and inferences about comparative effectiveness require controlled designs.

## Introduction

Mental health conditions, such as depression, anxiety, and stress-related difficulties, represent a leading cause of disability worldwide and are associated with substantial personal, social, and economic burden [1].

Barriers to access include limited availability of mental health professionals, long waiting times, geographic constraints, cost, and stigma, contributing to a persistent gap between mental health needs and service capacity [2,3]. In response to these challenges, digitally and remotely delivered psychological therapies have emerged as important strategies for expanding access to mental health care [3].

A growing body of research suggests that psychological therapy delivered via the internet and other remote modalities has shown clinical outcomes comparable with face-to-face treatment across common mental health conditions, while offering advantages in accessibility, flexibility, and scalability [4,5]. However, much of this evidence originates from structured research or trial settings, and less is known about how such interventions perform when delivered as part of routine clinical care.

In recent years, real-world evaluations of internet-delivered psychological services within employer- and insurance-based care settings have begun to address this gap. Early pragmatic evaluations of video-based blended care psychotherapy reported substantial symptom improvements under routine conditions [6]. Subsequent large-scale service evaluations replicated and extended these findings, showing that these outcomes could be maintained as services scaled to thousands of clients [7].

More recent evidence has strengthened both the comparative and the real-world case for remote delivery. A 2024 meta-analysis of 54 randomized trials found therapist-guided remote cognitive behavioral therapy comparable with in-person delivery (standardized mean difference=−0.02, 95% CI −0.12 to 0.07) [8], and routine-care data from the National Health Service showed equivalent outcomes for remotely and face-to-face delivered psychological treatment [9]. Large provider-run service evaluations in the United States have likewise reported substantial symptom change at scale [10,11]. This literature remains concentrated on structured or blended care models and on US and UK settings; however, we did not identify a peer-reviewed study published between 2020 and 2026 reporting isolable symptom outcomes for therapist-delivered synchronous video psychotherapy in Nordic or German-speaking routine or insurance-based care (as distinct from randomized or feasibility trials).

While these studies primarily examined structured blended care models, less is known about outcomes associated with routine online psychological therapy delivered without a standardized digital treatment curriculum, across a broad range of presenting concerns, and outside US and UK health systems. This study addresses this gap with a retrospective service evaluation of routine, curriculum-free video-delivered psychological therapy in a Danish insurance-based care setting, including perceived stress as an outcome alongside depression and anxiety, and with transparent reporting of attrition across the full service population. The aims were to describe (1) pre-post symptom change in depression, anxiety, and perceived stress, including rates of ≥50% symptom reduction and reliable change, and (2) patient-reported satisfaction.

## Methods

### Study Design

This study was conducted as a retrospective observational service evaluation based on routinely collected clinical outcome and service evaluation data. The evaluation reflects standard care delivery and did not involve any modification of treatment procedures, randomization, or comparison conditions. The primary aim was descriptive and quality-focused, namely, to characterize symptom change and patient satisfaction associated with routine online psychological therapy delivered under real-world conditions.

The data used in this evaluation were collected as part of routine clinical practice and quality assurance and were deidentified before being analyzed post hoc. No additional data were collected for research purposes. The evaluation included therapy courses initiated between September 1, 2024, and October 31, 2025. The evaluation is reported in accordance with the STROBE (Strengthening the Reporting of Observational Studies in Epidemiology) statement [12], informed by the RECORD extension for routinely collected health data [13]; the completed STROBE checklist is provided as Multimedia Appendix 1.

### Setting and Service Description

The service evaluated provides routine online psychological therapy within an insurance-based care setting. It operates through 2 insurance-based referral pathways. Clients access care through their insurance coverage and receive therapist-delivered individual psychological therapy via secure video consultation. Subproducts using different treatment modalities (internet-based cognitive behavioral therapy [CBT], counseling hotlines, and telephone-only therapy) were excluded to ensure a homogeneous sample of therapist-delivered video-based psychotherapy.

All therapy sessions and assessments were delivered via a secure, browser-based digital platform compliant with the European Union Digital Operational Resilience Act (DORA). The platform additionally supported asynchronous written communication between client and psychologist between sessions, and allowed psychologists to share digital content with the client as an optional adjunct to therapy. Neither the use of asynchronous messaging nor the sharing of digital content was a required element of the intervention.

All therapists delivering the intervention were psychologists authorized in accordance with Danish national standards and had completed formal clinical practice training (Danish: *praksisuddannelse*). Therapists were required to have documented previous experience applying CBT principles, but no formal postgraduate specialist qualification was required. Before onboarding to the platform, all therapists completed a structured training protocol covering service-specific clinical method, expectations regarding therapeutic conduct, and professional-quality standards.

Therapy was delivered as individual video sessions, typically lasting approximately 45 minutes. Clients and therapists connected from locations of their own choice, without constraints on physical setting. Session scheduling was flexible, determined jointly by client needs and therapist availability, with no predefined frequency.

The therapeutic process was anchored in CBT principles. Beyond the standardized baseline symptom screening described further in this study (refer to Measures section), no standardized intake template, case-formulation document, or structured treatment-planning artifact was imposed at the service level. Specific interventions—ranging from cognitive and behavioral techniques to psychoeducation, stress management, and supportive therapeutic dialogue—were selected by the treating psychologist based on the client’s presenting concerns, treatment goals, and the psychologist’s evolving case formulation. Psychologists were expected to incorporate between-session assignments, with content selected at the psychologist’s discretion and tailored to the individual client.

No predefined minimum or maximum number of sessions was imposed; course length was determined jointly by client and psychologist based on clinical need, within a service model designed to provide short-term psychological therapy. Session attendance data (sessions held, canceled, or not attended) were routinely collected at the service level and are reported below as a descriptive measure of intervention delivery. Booking requests that were canceled before reaching an accepted state (ie, before a video session room was created) were excluded from planned-session counts.

No material changes to the service model including platform architecture, referral pathways, therapist onboarding protocol, baseline-screening procedures, or therapist qualification requirements occurred during the data collection period (September 1, 2024, to October 31, 2025).

### Quality Assurance and Fidelity

Routine outcome data (baseline and end-of-treatment symptom scores) were used for service-level monitoring only; individual-client symptom trajectories were not routinely surfaced to the treating psychologist during care. Biannual audits of clinical documentation were conducted by we.care (We.Care Health ApS), with the audit findings being followed up on at the group and individual therapist level where indicated. No formal individual clinical supervision was mandated at the service level during the study period.

### Participants

The study population consisted of clients aged 15 years and older who initiated online psychological therapy within the service and completed at least 1 baseline assessment and 1 end-of-treatment assessment following course closure. Access to the service required acceptance of therapy delivered via an online platform. Inclusion criteria for the analyses of this study were the availability of matched baseline and end-of-treatment outcome data on all 3 primary symptom measures (9-item Patient Health Questionnaire [PHQ-9], 7-item Generalized Anxiety Disorder [GAD-7], and 10-item Perceived Stress Scale [PSS-10]). Clients meeting these criteria are hereafter referred to as the paired-assessment sample; the term denotes availability of both assessments and does not imply a clinically defined treatment completion. For clients with multiple treatment episodes, only the earliest course was retained to ensure independence of observations. Clients included were not selected based on specific diagnoses; rather, presenting concerns were heterogeneous, reflecting routine clinical practice. Exclusion criteria were active suicidality, homicidality, psychiatric disorders that were not stabilized by medication, and substance abuse disorders. Written consent to participate in treatment was obtained for all clients included in this service evaluation. The service is offered to clients aged 15 years and older, consistent with The Danish Health Act §17, which grants autonomous consent to health care from this age. Clients younger than 15 years were not eligible for the service and were therefore not included.

Exclusion criteria for the analyses were limited to missing or incomplete outcome data preventing calculation of change scores.

### Data Linkage

Baseline and end-of-treatment screening responses were linked to course records using a client-entered numeric identifier. A tiered matching strategy was applied as follows: (1) exact match after digit extraction, (2) unambiguous fuzzy match at Levenshtein distance of 1 [14] (accepted only when a single database candidate existed), and (3) manual review and correction by the authors for remaining unmatched entries. For clients with multiple treatment episodes, screenings were assigned to the temporally nearest course using Voronoi midpoint boundaries between consecutive courses [15]; only the earliest course per client was retained for analysis (counts of repeat-client courses excluded under this rule are reported in the Results section). Within the analytical sample, every baseline screening preceded the first held session, and every end-of-treatment screening followed the last held session. Baseline screenings were completed a median of 5.9 (IQR 3.5-8.8) days before the first session; end-of-treatment screenings a median of 1.2 (IQR 0.1-6.1) days (95% within 54 days) after the last session. A sensitivity analysis was conducted comparing effect sizes across matching tiers (tier 1 only, tier 1+2, and tier 1+2+3) to verify that matching methodology did not bias results. The linkage procedure, tier counts, and manual-correction volume are visualized in a linkage diagram (Multimedia Appendix 2).

### Measures

#### Clinical Outcome Measures

Symptoms of depression, anxiety, and perceived stress were assessed using standardized self-report instruments routinely administered at treatment initiation and at the final available assessment as part of clinical outcome monitoring. Baseline screening administration differed by referral pathway; mandatory before the first therapy session in one pathway, and delivered by platform message at registration (encouraged but not required) in the other. Of the 1221 clients in the paired-assessment sample, 791 (64.8%) entered via the pathway in which baseline screening was encouraged at registration but not required, and 430 (35.2%) via the pathway with mandatory presession screening. The end-of-treatment screening link was dispatched automatically when the treating psychologist closed the course, regardless of closure reason (mutually agreed completion, referral to another provider, client-initiated discontinuation, or prolonged nonresponse). Inclusion in the paired-assessment sample therefore reflects return of both baseline and end-of-treatment screenings under these administration conditions, rather than a clinically defined treatment-completion criterion.

Depressive symptoms were measured using the PHQ-9, a 9-item self-report questionnaire assessing the frequency of depressive symptoms over the past 2 weeks. Total scores range from 0 to 27, with higher scores indicating greater symptom severity [16].

Anxiety symptoms were assessed using the GAD-7, a 7-item self-report measure evaluating anxiety symptom severity over the past 2 weeks. Total scores range from 0 to 21, with higher scores reflecting greater anxiety severity [17].

Perceived stress was measured using the PSS-10, a 10-item self-report instrument assessing the degree to which situations in one’s life are appraised as stressful. Total scores range from 0 to 40, with higher scores indicating higher perceived stress [18].

#### Presenting Concerns

Presenting concerns were recorded through pathway-specific routine processes. In the encouraged-screening pathway, each course carried a presenting-concern category from a fixed 28-category service taxonomy, reflecting the reason for referral as recorded by the referring organization’s health professionals; these data were available for 706 of 791 (89.3%) courses. In the mandatory-screening pathway, therapy is in most cases preceded by an assessment consultation with an assessing psychologist, who recorded a presenting-concern category from a separate 12-option taxonomy, confirming or revising the referral reason; these data were available for 377 of the 379 (99.5%) courses entering through that assessment. Assessment records were assigned to courses as the temporally closest record preceding course creation. A small direct-booking subgroup within this pathway (51 courses) enters therapy on a referral without the assessment consultation, and its referral reason is not captured in a structured field. In total, structured presenting-concern data were available for 1083 of 1221 (88.7%) courses. Categories in both taxonomies are presenting-concern labels, not clinical diagnoses; no structured diagnostic assessment was performed as part of routine care. The 2 taxonomies are reported separately, as recorded, without cross-pathway harmonization, and semantically adjacent labels (eg, anxiety and anxiety symptoms) were not collapsed.

#### Patient-Reported Satisfaction

Patient-reported satisfaction with care was assessed at the end-of-treatment assessment using 3 single-item questions rated on a 10-point Likert scale (1=not at all satisfied and 10=completely satisfied). Clients were asked to rate their satisfaction with (1) contact and service, (2) the psychologist, and (3) the technical platform used to deliver care. In addition, a single-item measure of recommendation likelihood was administered (“How likely are you to recommend we.care to someone you know?”), rated on the same 10-point scale. These items were collected as part of routine service evaluation to assess patient satisfaction and perceived service quality.

#### Demographic and Treatment Variables

Age was derived from date of birth registered by the client at sign-up on the platform. Gender information was available for 93.9% of the analytical sample. Treatment characteristics included the number of completed therapy sessions and treatment duration, defined as the number of days between the first and last completed session. At course closure, the treating psychologist recorded a closure status indicating whether the client was assessed as having completed treatment within the framework of the course (recorded as successful or unsuccessful). This field was available for 99.8% (1219/1221) of the paired-assessment sample and 98.4% (5782/5876) of all courses. It is a single binary judgement recorded by the treating therapist at closure. No operational criteria for “successful” were specified at the service level, interrater reliability was not assessed, and the field was neither independently verified nor recorded blind to the course of treatment. It is not a symptom outcome and is reported descriptively in the attrition analysis only.

### Statistical Analyses

The primary outcome set (PHQ-9, GAD-7, and PSS-10), descriptive analyses, paired-sample *t* tests for pre-post change, Cohen *d* as the within-sample effect-size metric, and the clinical-significance framework (≥50% symptom reduction and below cutoff classification) were prespecified in an internal project brief dated November 6, 2025, before data extraction. The screening-to-course matching strategy (tier 1, 2, or 3 with Voronoi-based assignment for repeat clients), the reliable change index (RCI) analysis, the baseline-elevated subgroup analysis, the attrition comparisons across assessment-completion groups, and the sensitivity analysis across matching tiers were developed during analysis in response to the observed data structure and are reported transparently in the Results section. The therapist-clustering analysis, the inverse-probability–weighted and zero-change sensitivity analyses, the end-of-treatment assessment-window sensitivity analysis, the exploratory moderator model, and the presenting-concern characterization were added in response to peer review.

Descriptive statistics were used to summarize demographic characteristics, treatment characteristics, and baseline symptom severity. Continuous variables are reported as means and SDs, and categorical variables as frequencies and percentages.

Changes in symptom scores from baseline to the final available assessment were examined using paired sample *t* tests for each outcome measure (PHQ-9, GAD-7, and PSS-10). Given the large sample size, paired *t* tests were considered sufficiently robust to modest deviations from normality. Statistical tests were used descriptively to characterize within-sample change rather than for confirmatory hypothesis testing.

Within-sample effect sizes were calculated using Cohen *d*, defined as the mean change score divided by the SD of baseline scores, with 95% CIs computed via the noncentral t distribution approximation [19]. Effect sizes are reported to describe the magnitude of observed change under routine care conditions and should not be interpreted as estimates of treatment-specific efficacy. Baseline-SD standardization was retained for comparability with previous real-world evaluations reporting this metric [6,7] and because it places within-subject effect sizes on the same metric as between-group designs [20]. Because the choice of standardizer materially affects within-subject effect sizes, the change-score-standardized effect size Cohen *d_z_* (mean change divided by the SD of change scores, which incorporates the pre-post correlation) is reported alongside Cohen *d* for all measures [21].

Because clients were nested within therapists, and nesting violates the independence assumption of unadjusted tests [22], change scores for each outcome measure were additionally analyzed using linear mixed-effects models with a random intercept for the treating therapist, defined as the modal therapist across held sessions. The intraclass correlation coefficient (ICC) was derived from the estimated variance components. Because even small ICCs can inflate SEs [23], mean change estimates and CIs were compared across the following 3 approaches: the mixed model, cluster-robust SEs, and the unclustered paired *t* test.

An exploratory moderator model was fitted for each measure, regressing change scores on standardized baseline severity, age, gender, number of held sessions, treatment duration, and referral pathway, with a random intercept for therapist. Clients with missing age or gender were excluded from this model (complete-case basis; n reported in Table S9 in Multimedia Appendix 3). Estimates are reported descriptively with 95% CIs and without adjustment for multiplicity, and are not interpreted causally.

To assess sensitivity of the results to attrition, two analyses were conducted. First, inverse-probability weighting (IPW) was applied as a sensitivity analysis, following recommendations to prefer a parsimonious weight model restricted to baseline covariates and to treat IPW as a robustness check rather than a primary estimator [24]. The probability of returning the end-of-treatment screening was modeled among baseline-screened clients by logistic regression on age, gender, baseline PHQ-9, GAD-7, and PSS-10 scores, and referral pathway; the number of sessions and treatment duration are postbaseline treatment-process variables and were therefore excluded from the primary weight model and examined only in a secondary weight model. Weights were stabilized and trimmed at the 1st and 99th percentiles [25], and weighted mean change and effect sizes were recomputed. The weight model was fitted on covariate-complete cases: 1142 of 1221 paired-assessment clients and 2197 of 2382 baseline-only clients (3339 of 3603). These weights target the population of baseline-screened clients under a missing-at-random-given-covariates assumption; attrition at the no-screening stage (n=1715) could not be modeled, as no baseline scores exist for these clients. Second, a zero-change analysis imputed a change of zero for every baseline-screened client without an end-of-treatment assessment. This is a reference scenario rather than a worst case or a lower bound, as nonassessed clients could have deteriorated. As a further robustness check on linkage and assessment timing, all effect sizes were recomputed with the paired-assessment sample restricted to courses whose end-of-treatment screening was completed within 60 days of the last held session, the intended end-assessment window in the linkage configuration.

A ≥50% reduction from baseline to final score on each outcome measure was reported as a descriptive response criterion, following previous real-world evaluations of online psychological therapy [6,7]. This criterion is pragmatic and is not a validated clinical-significance threshold. It is used to describe the magnitude of symptom reduction and is not interpreted as evidence of clinical significance; this applies in particular to the PSS-10, for which no validated clinical cutoff and no generalizable minimal important change are established (refer to Limitations section). In addition, symptom status at the end-of-treatment assessment was evaluated relative to established clinical cutoff thresholds for the primary depression and anxiety outcomes (PHQ-9<10 [16]; GAD-7<8 [17,26,27]). No equivalent analysis is reported for the PSS-10, as no validated Danish clinical cutoff is established for this instrument; PSS-10 change is instead reported continuously using means, effect sizes, and the RCI. These analyses were conducted for the full sample; below-cutoff classification was additionally evaluated among clients with elevated baseline scores on the respective measure (PHQ-9≥10; GAD-7≥8). Reliable change was assessed using the RCI [28]. The RCI was calculated as (prescore minus postscore) divided by the SE of the difference, where the SE was derived from the baseline SD and published test-retest reliability coefficients (PHQ-9=0.84 [16], GAD-7=0.83 [17], and PSS-10=0.87 [29]). Clients with RCI>1.96 were classified as reliably improved, those with RCI<–1.96 as reliably deteriorated, and the remainder as showing no reliable change. The RCI indicates whether an individual’s observed change exceeds the measurement error of the instrument. It is a measurement-precision criterion and does not establish clinical significance; reliable-improvement rates are reported on that basis.

An attrition analysis compared following three groups defined by assessment completion: (1) clients with at least 1 held session but no matched baseline screening (“no screening”), (2) clients with a matched baseline screening but no end-of-treatment screening (“start only”), and (3) clients with both baseline and end-of-treatment screenings (the paired-assessment sample). Continuous variables (age, number of sessions, treatment duration, and baseline symptom severity) were compared between the start-only and paired-assessment groups using independent samples *t* tests (Welch *t* test, not assuming equal variances). Categorical variable gender was compared between the same two groups using chi-square tests. The no-screening group was reported descriptively without statistical tests, as this group lacked baseline symptom data. Gender was categorized as female, non-female, or missing for this comparison.

All analyses were conducted on fully deidentified data using Python (version 3.14.3; Python Software Foundation) with *pandas* (version 2.3.3), *NumPy* (version 2.4.3), *SciPy* (version 1.17.1), and *statsmodels* (version 0.14.6). All statistical tests were 2-sided with a significance level of α=.05.

### Ethical Considerations

This evaluation constitutes retrospective quality assurance and service evaluation of routinely collected clinical data, not a health-science research project. The determination that a research ethics committee review was not required was made by the first author (TO), chief psychologist at we.care and the clinician with overall responsibility for the service, on the basis of the Danish Act on Research Ethics Review of Health Research Projects and Health Data Science Research Projects (komitéloven) [30] and the notification guidance issued by the Danish National Center for Ethics [31]. Two independent grounds apply. First, quality assurance and service evaluation activities, which do not aim to generate new knowledge about the value of a treatment, fall outside the definition of a health-science research project and are not subject to notification [31]. Second, §14(2) of the Act requires notification of health-science questionnaire surveys and register-based research projects only where the project involves human biological material [30]; the present evaluation used questionnaire and administrative data only, with no human biological material, no intervention, no randomization, and no deviation from routine care. Notification would therefore not have been required even had the activity been classified as research. Because the activity is exempt by statute, no application was submitted and no written determination or approval was issued by any committee or authority; none is required for an activity of this character under Danish law. TO likewise authorized the secondary analysis on behalf of we.care as data controller. Processing was carried out for quality assurance and service evaluation within the organization that delivered the care, in accordance with the EU General Data Protection Regulation (GDPR) and the Danish Data Protection Act; no data were transferred outside we.care.

The same statutory basis applies to clients aged 15-17 years (n=21, 1.7% of the analytical sample). The notification exemption is not age-dependent, and no separate research consent or parental consent was required for the use of their deidentified routine data. Under §17 of the Danish Health Act [32], patients aged 15 years and older may consent to health care themselves, and all clients in this age band consented to treatment on that basis.

Written consent to participate in treatment was obtained from all clients as part of routine clinical practice. The data were deidentified before analysis; identifiers used during deterministic record linkage (screening-to-course matching) were not retained in the analytical dataset, and data handling was conducted in accordance with the EU GDPR and the Danish Data Protection Act. Clients received no compensation; all assessments were completed as part of routine care.

### Data Sharing

Aggregate data corresponding to the main-text tables and figures and to the sensitivity and supplementary analyses, a codebook, and the Python analysis code are deposited at the Open Science Framework (OSF [33]) under a CC-BY 4.0 license. Individual-level data are not shared; the Data Availability statement specifies what the deposit supports.

## Results

### Study Population and Treatment Characteristics

A total of 1221 clients with matched baseline and end-of-treatment assessments on all 3 outcome measures were included in the service evaluation. Clients completed a mean of 4.7 (SD 1.67) therapy sessions, with a median of 4 (IQR 4-6; range 1-12) sessions. The mean duration of care, defined as the number of days between the first and last completed session, was 62.7 (SD 40.4) days. The mean time from course creation to first session was 6.6 (SD 5.4) days. Demographic and treatment characteristics are summarized in Table 1.

The sample was predominantly of working age (mean age 39.6, SD 12.2 y) and 61.8% (755/1221) female (Table 1).

Baseline symptom severity indicated moderate levels of distress across outcome measures. Mean baseline scores were 12.47 (SD 5.19) for depressive symptoms (PHQ-9), 12.21 (SD 4.73) for anxiety symptoms (GAD-7), and 23.18 (SD 5.61) for perceived stress (PSS-10).

Presenting concerns, recorded for 706 of 791 (89.3%) courses in the encouraged-screening pathway (refer to Methods section), most frequently comprised stress (160/706, 22.7%), being a relative of someone seriously ill (96/706, 13.6%), anxiety symptoms (72/706, 10.2%), depression (69/706, 9.8%), and anxiety (68/706, 9.6%). In the mandatory-screening pathway, assessment-recorded concerns (377 of 379 assessed courses, 99.5%) were dominated by psychological stress (131/377, 34.7%) and poor well-being and stress reaction (117/377, 31%; Table 1; refer to Table S5 in Multimedia Appendix 3 for full distributions).

Session attendance across the analytical sample was high. A total of 6781 sessions were planned, of which 5736 (84.6%) were held, 1005 (14.8%) were canceled or rescheduled, 27 (0.4%) were recorded as no-shows, and 13 (0.2%) were not held due to platform downtime. On a per-course basis, a mean of 87.2% (SD 14.3%) of planned sessions were held, with a median of 4 (IQR 4-6) sessions held per course. The platform did not distinguish rescheduled bookings from permanent cancellations or record the initiating party. The median cancellation lead time was 2.0 (IQR 1.0-5.8) days, with only 6.1% (61/1005) of cancellations occurring less than 2 hours before the scheduled session; 49.4% (603/1221) of courses had no cancellations. The reported cancellation rate should therefore be interpreted as an upper bound on sessions permanently lost to cancellation.

**Table 1:** Baseline demographics and treatment characteristics (N=1221).

Characteristic			Value
Age (y), mean (SD)			39.55 (12.17)
Age (y), median (IQR)			38.0 (31.0–49.0)
Age group (y), n (%)			
	<18		21 (1.7)
	18-25		120 (9.8)
	26-30		135 (11.1)
	31-40		362 (29.6)
	41-50		251 (20.6)
	51-60		201 (16.5)
	61+		52 (4.3)
	Missing		79 (6.5)
Gender, n (%)^a^			
	Female		755 (61.8)
	Nonfemale		392 (32.1)
	Missing		74 (6.1)
Held sessions, mean (SD)			4.7 (1.67)
Held sessions, median (IQR)			4.0 (4.0–6.0)
Treatment duration (d), mean (SD)			62.69 (40.43)
Presenting concern (referral-derived), n (%)^b^			
	Stress (including work-related stress)		160 (22.7)
	Relative of someone who is seriously ill		96 (13.6)
	Anxiety symptoms		72 (10.2)
	Depression		69 (9.8)
	Anxiety		68 (9.6)
	Depressive symptoms		66 (9.3)
	Adjustment disorder		57 (8.1)
	Acute stress reaction		47 (6.7)
	People affected by serious and/or disabling illness		32 (4.5)
	Termination or dismissal from work		8 (1.1)
	Well-being or poor well-being		6 (0.8)
	Abortion		5 (0.7)
	Other (combined, each n<5)		20 (2.8)
Presenting concern (assessment-derived), n (%)^c^			
	Psychological stress	131 (34.7)	
	Poor well-being and stress reaction	117 (31)	
	Relationship difficulties	31 (8.2)	
	Relative of a person with mental or physical illness	26 (6.9)	
	Anxiety		26 (6.9)
	Depression		21 (5.6)
	Death in the family		12 (3.2)
	Serious illness		8 (2.1)
	Other (combined, each n<5)		5 (1.3)

^a^Gender categorized as female, nonfemale, or missing. Nonfemale includes male, nonbinary, and other gender identities; categories were collapsed to prevent reidentification of small subgroups (n<5).

^b^Referral-derived presenting-concern categories from a fixed 28-category taxonomy (recorded at course level; 1 category per course; not diagnoses); the category reflects the reason for referral recorded by the referring organization’s health professionals, not the treating psychologist. Coverage: 706 of 791 encouraged screening pathway courses (89.3%); courses on the mandatory-screening pathway carry no such categories.

^c^Assessment-derived presenting-concern categories from a fixed 12-category taxonomy (recorded once per course by the assessing psychologist at the initial assessment consultation; one category per course; not diagnoses), recorded on the mandatory-screening pathway. Coverage: 377 of 379 mandatory-screening-pathway courses (99.5%). A direct-booking subgroup (n=51) bypasses the assessment consultation by design and records no such category; it is excluded from this denominator rather than counted as missing.

An attrition analysis compared 3 groups based on assessment completion (Table 2)—clients with at least 1 session but no baseline screening (n=1715), clients with a baseline screening but no end-of-treatment screening (n=2382), and clients with both assessments (the paired-assessment sample; n=1221). Statistical comparisons were conducted between the start-only and paired-assessment groups. The no-screening group is reported descriptively only. Paired-assessment clients were slightly older (mean 39.6, SD 12.2 y) than start-only clients (mean 38.2, SD 11.6 y; t_2198.7_=−3.04; *P*=.002), completed more sessions (mean 4.7, SD 1.67 vs mean 4.2, SD 1.84; t_2675.9_=−8.66; *P*<.001), and had longer treatment duration (mean 62.7, SD 40.4 vs mean 55.8, SD 44.5 days; t_2675.2_=−4.67; *P*<.001). The proportion of female clients did not differ significantly between the paired-assessment sample (755/1221, 61.8%) and start-only clients (1400/2382, 58.8%; *χ*²_2_=5.7, *P*=.06).

Baseline symptom severity did not differ significantly between the start-only group and the paired-assessment sample (all *P*≥.48; Table 2), suggesting that attrition was not driven by initial symptom severity.

**Table 2:** Comparison of participant groups by attrition stage.

Characteristic			No screening^a^ (n=1715)		Start only (n=2382)		Paired assessments (n=1221)		*t* test (*df*) or chi-square (*df*)			*P* value	
Age (y), mean (SD)			37.3 (12.17)		38.23 (11.59)		39.55 (12.17)		−3.04 (2198.7)^b^			.002	
Held sessions, mean (SD)			4.21 (1.78)		4.17 (1.84)		4.7 (1.67)		−8.66 (2675.9)^b^			<.001	
Treatment duration (d), mean (SD)			54.72 (41.59)		55.81 (44.47)		62.69 (40.43)		−4.67 (2675.2)^b^			<.001	
PHQ-9^c^ baseline, mean (SD)			—^d^		12.59 (5.41)		12.47 (5.19)		0.62 (2559.4)^b^			.54	
GAD-7^e^ baseline, mean (SD)			—		12.32 (4.94)		12.21 (4.73)		0.61 (2569.1)^b^			.54	
PSS-10^f^ baseline, mean (SD)			—		23.32 (5.92)		23.18 (5.61)		0.70 (2579)^b^			.48	
Gender, n (%)										5.7 (2)^g^			.06
	Female	945 (55.1)		1400 (58.8)		755 (61.8)							
	Nonfemale	567 (33.1)		857 (36)		392 (32.1)							
	Missing	203 (11.8)		125 (5.2)		74 (6.1)							

^a^No-screening group reported descriptively (no statistical test).

^b^Independent samples *t* test (Welch, unequal variances) comparing start-screening-only group with the paired-assessment group; *df* reported per the Welch-Satterthwaite approximation.

^c^PHQ-9: 9-item Patient Health Questionnaire.

^d^Not available.

^e^GAD-7: 7-item Generalized Anxiety Disorder.

^f^PSS-10: 10-item Perceived Stress Scale.

^g^Pearson chi-square test comparing start-screening-only group with the paired-assessment group.

Therapist-recorded closure status (refer to Methods section) provided descriptive, nonindependent context on noncompletion of assessments. Within the paired-assessment sample, 1198 of 1221 (98.1%) courses were closed as successful and 19 (1.6%) as unsuccessful. Among start-only clients, 85.5% (2036/2382) of courses were closed as successful and 12.7% (302/2382) as unsuccessful (41/2382, 1.7% missing); among no-screening clients, 86.8% (1487/1713) and 11.4% (196/1713), respectively (27/1713, 1.6% missing). Outcomes were not stratified by closure status, as the 19 unsuccessful closures in the paired-assessment sample preclude stable stratified estimates. The full distribution by cohort is provided in Table S12 in Multimedia Appendix 3.

Figure 1 presents a flow diagram of participant inclusion. Of 5876 courses created in the study period, 5798 had a therapist assigned, and 5562 had at least 1 held session. Start screening data were matched for 3690 of these courses. Of these, 1329 had both start and end screenings paired. Within this paired-screening cohort, only the earliest course per client was retained to ensure independence of observations, excluding 9 repeat-client courses. After requiring complete scores on all three outcome measures, 1264 clients remained. To ensure temporal validity of pre-post comparisons, courses where the baseline screening was submitted after the first held session or the end screening before the last held session were excluded (n=43), yielding a final analytical sample of 1221 clients. For the attrition analysis (Table 2), the same earliest per-client rule was applied across the full held-session population: 227 clients had more than one course in the study window, and 244 nonearliest courses were excluded, yielding 5318 unique clients classified into 3 assessment-completion groups.

**Figure 1:**
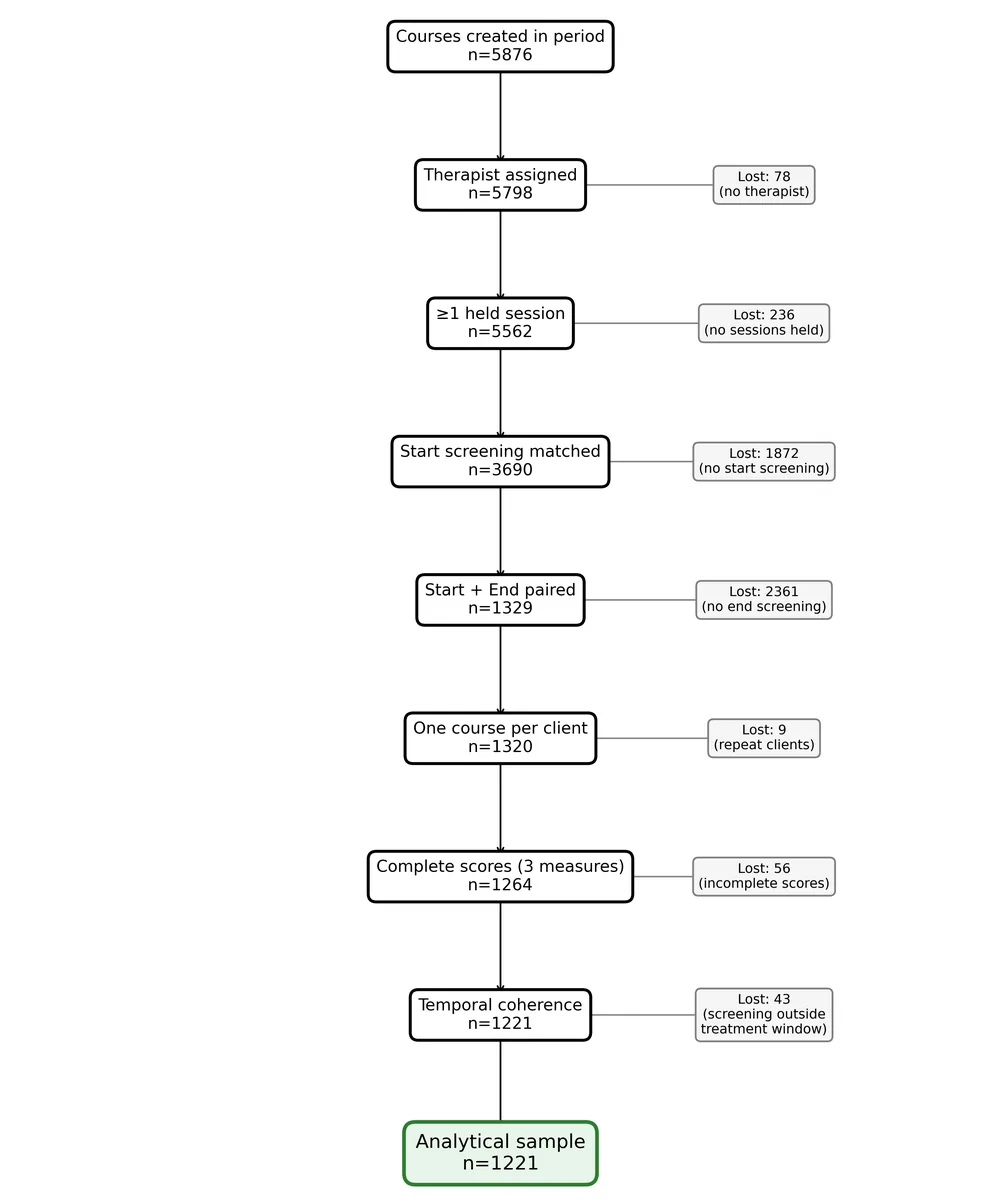
Flow diagram of participant inclusion and exclusion. Of 5876 therapy courses created between September 1, 2024, and October 31, 2025, sequential criteria were applied to yield the paired-assessment analytical sample (n=1221). Exclusion counts at each step indicate courses removed at that stage only.

### Changes in Depression, Anxiety, and Perceived Stress

Reductions in symptom scores were observed from baseline to the final available assessment across all outcome measures (Table 3).

Mean reductions were 6.47 points (49.4%) on the PHQ-9, 7.03 points (54.2%) on the GAD-7, and 8.20 points (33.8%) on the PSS-10; all changes were statistically significant, with large within-sample effect sizes (Table 3).

Effect sizes are reported to describe the magnitude of observed within-sample change under routine care conditions and should not be interpreted as estimates of treatment-specific efficacy. Change score standardized effect sizes were of similar magnitude: Cohen *d_z_*=1.25 (95% CI 1.18-1.33) for PHQ-9, Cohen *d_z_*=1.37 (95% CI 1.30-1.45) for GAD-7, and Cohen *d_z_*=1.20 (95% CI 1.12-1.27) for PSS-10 (Table 3). The distributions of symptom scores at baseline and at the end-of-treatment assessment are presented in Figure 2.

**Table 3:** Pre-post symptom changes and within-sample effect sizes (N=1221).

Measure	Participants, n	Start, mean (SD)	End, mean (SD)	ΔMean (SD)	*t* test (*df*)	Cohen *d* (95% CI)^a^	Cohen *d*_*z*_ (95% CI)^b^	*P* value
PHQ-9^c^	1221	12.47 (5.19)	6.0 (4.64)	6.47 (5.18)	43.70 (1220)	1.247 (1.172-1.322)	1.251 (1.176-1.325)	<.001
GAD-7^d^	1221	12.21 (4.73)	5.18 (4.12)	7.03 (5.12)	48.01 (1220)	1.488 (1.406-1.569)	1.374 (1.296-1.452)	<.001
PSS-10^e^	1221	23.18 (5.61)	14.98 (6.53)	8.2 (6.85)	41.84 (1220)	1.462 (1.381-1.542)	1.197 (1.124-1.271)	<.001

^a^Cohen *d* = mean(change)/SD(baseline), 95% CI via noncentral t distribution. Paired-samples *t* test, *df* = N − 1.

^b^Cohen *d*_z_ = mean(change)/SD(change), paired or within-subject standardizer [20,21]. Reported alongside baseline-SD Cohen *d* for comparability with prior effectiveness studies.

^c^PHQ-9: 9-item Patient Health Questionnaire.

^d^GAD-7: 7-item Generalized Anxiety Disorder.

^e^PSS-10: 10-item Perceived Stress Scale.

**Figure 2:**
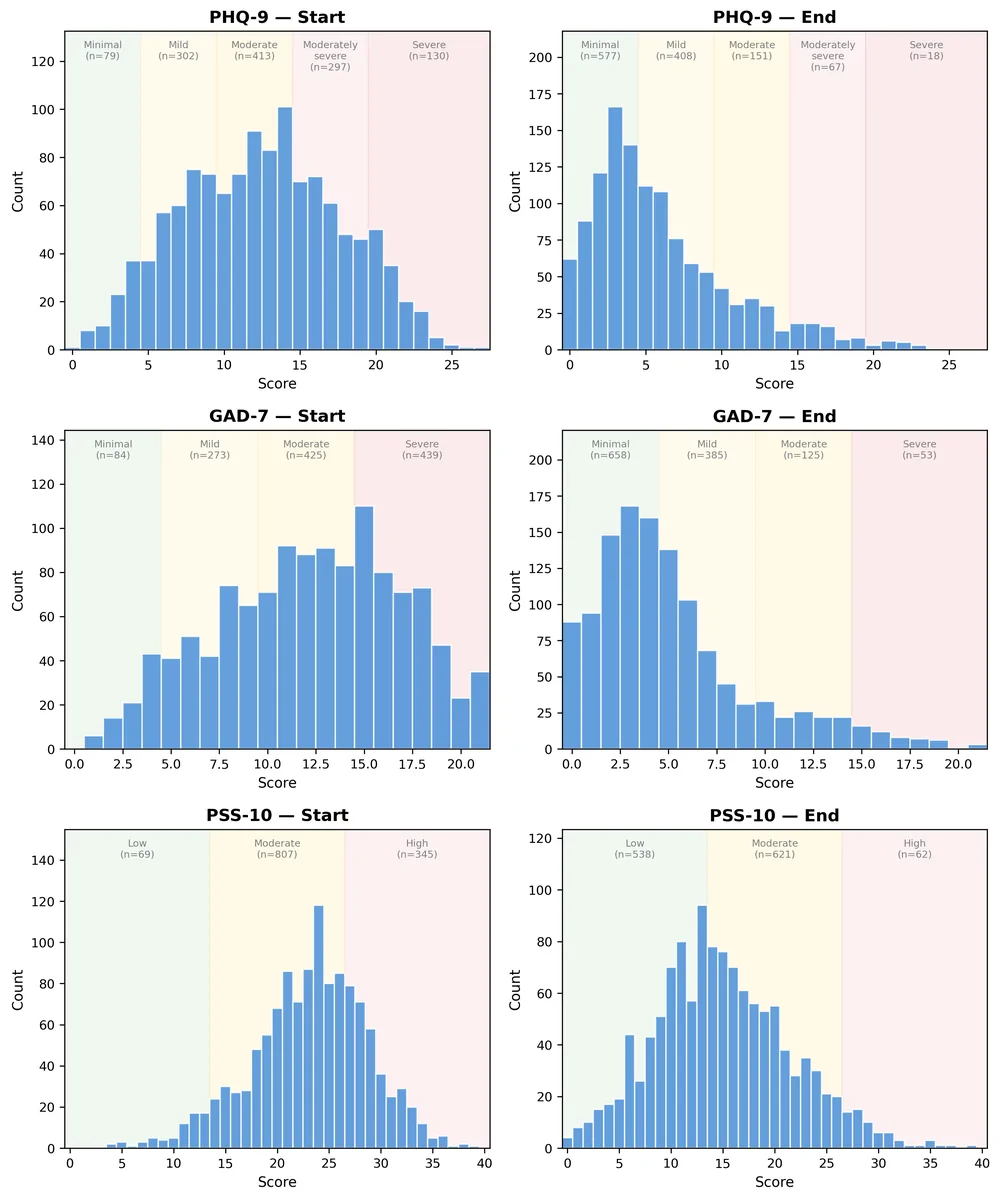
Distribution of symptom scores at baseline and at the end-of-treatment assessment (N=1221) for depression (PHQ-9), anxiety (GAD-7), and perceived stress (PSS-10). Shaded regions indicate severity bands: PHQ-9—minimal (0-4), mild (5-9), moderate (10-14), moderately severe (15-19), severe (20-27); GAD-7—minimal (0-4), mild (5-9), moderate (10-14), severe (15-21); PSS-10—low (<14), moderate (14-26), high (≥27). The PSS-10 bands are distributional norm-based reference ranges, not validated clinical cut points. Client counts within each band are reported in the panel headers. GAD-7: 7-item Generalized Anxiety Disorder; PHQ-9: 9-item Patient Health Questionnaire; PSS-10: 10-item Perceived Stress Scale.

### Symptom Reduction and Below-Cutoff Status

A ≥50% reduction from baseline to final score was observed in a substantial proportion of clients; reliable change analysis showed a consistent pattern (Tables 4-7). The ≥50% reduction criterion is a descriptive response criterion, not a validated clinical-significance threshold; this applies in particular to the PSS-10. In Table 4, ≥50% reduction requires a baseline score >0 (a zero baseline cannot fall by 50%); clients with baseline=0 are excluded from the denominator for all 3 measures (n excluded: PHQ-9=1, GAD-7=0, and PSS-10=0). In Tables 5 and 6, no validated clinical cutoff exists for the PSS-10, which is therefore reported continuously and via the reliable change index only (refer to Methods section).

A total of 60.7% (95% CI 58.0-63.4) of clients achieved a ≥50% reduction in depressive symptoms on the PHQ-9, 67.5% (95% CI 64.8-70.1) achieved this level of improvement on the GAD-7, and 30.8% (95% CI 28.3-33.4) achieved a ≥50% reduction on the PSS-10.

When symptom status at the end-of-treatment assessment was evaluated relative to established clinical cutoff thresholds, 80.7% (95% CI 78.4-82.8) of clients scored below the clinical cutoff for depression (PHQ-9<10) and 79.2% (95% CI 76.8-81.4) scored below the cutoff for anxiety (GAD-7<8).

Most clients presented with elevated baseline symptoms on at least 1 measure (PHQ-9≥10 and/or GAD-7≥8; n=1070, 87.6% of the paired-assessment sample). Among clients elevated at baseline on the respective measure, 74.2% (95% CI 71.1-77.0) of those with baseline PHQ-9≥10 (n=840) scored below the depression cutoff at the end-of-treatment assessment, and 75.8% (95% CI 73.0-78.3) of those with baseline GAD-7≥8 (n=1003) scored below the anxiety cutoff.

**Table 4:** Clients with a ≥50% symptom reduction from baseline to the end-of-treatment assessment.

Measure	Participants, n	Participants with ≥50% reduction, n	%^a^ (95% CI)
PHQ-9^b^	1220	741	60.7 (58.0-63.4)
GAD-7^c^	1221	824	67.5 (64.8-70.1)
PSS-10^d^	1221	376	30.8 (28.3-33.4)

^a^All percentages are shown with Wilson score 95% CIs.

^b^PHQ-9: 9-item Patient Health Questionnaire.

^c^GAD-7: 7-item Generalized Anxiety Disorder.

^d^PSS-10: 10-item Perceived Stress Scale.

**Table 5:** Clients scoring below the clinical cutoff at the end-of-treatment assessment.

Measure	Cutoff	Participants, n	Participants below cutoff, n	%^a^ (95% CI)
PHQ-9^b^	<10	1221	985	80.7 (78.4-82.8)
GAD-7^c^	<8	1221	967	79.2 (76.8-81.4)

^a^All percentages are shown with Wilson score 95% CIs.

^b^PHQ-9: 9-item Patient Health Questionnaire.

^c^GAD-7: 7-item Generalized Anxiety Disorder.

**Table 6:** Clients scoring below the clinical cutoff at the end-of-treatment assessment among those with elevated baseline scores.

Measure	Baseline criterion	Participants (elevated at baseline), n	Participants below cutoff at end, n	%^a^ (95% CI)
PHQ-9^b^	≥10	840	623	74.2 (71.1-77)
GAD-7^c^	≥8	1003	760	75.8 (73-78.3)

^a^All percentages are shown with Wilson score 95% CIs.

^b^PHQ-9: 9-item Patient Health Questionnaire.

^c^GAD-7: 7-item Generalized Anxiety Disorder.

**Table 7:** Reliable change classification by measure^a^.

Measure	Test-retest *r*^b^	Participants, n	Improved		No change		Deteriorated	
			n (%)	95% CI	n (%)	95% CI	n (%)	95% CI
PHQ-9^c^	0.84	1221	688 (56.3)	53.5-59.1	524 (42.9)	40.2-45.7	9 (0.7)	0.4-1.4
GAD-7^d^	0.83	1221	747 (61.2)	58.4-63.9	461 (37.8)	35.1-40.5	13 (1.1)	0.6-1.8
PSS-10^e^	0.87	1221	794 (65)	62.3-67.7	405 (33.2)	30.6-35.9	22 (1.8)	1.2-2.7

^a^Reliable change index (RCI) = (pre − post)/SE_diff, where SE_diff = √(2) × SD_baseline × √(1−r). Reliably improved if RCI>1.96; deteriorated if RCI<−1.96.

^b^Test-retest reliability coefficients from published validation studies.

^c^PHQ-9: 9-item Patient Health Questionnaire.

^d^GAD-7: 7-item Generalized Anxiety Disorder.

^e^PSS-10: 10-item Perceived Stress Scale.

### Reliable Change

Reliable change analysis (Tables 4-7) indicated that 56.3% (688/1221) of clients achieved reliably improved depression scores (RCI>1.96), with only 0.7% (9/1221) showing reliable deterioration. For anxiety, 61.2% (747/1221) showed reliable improvement with 1.1% (13/1221) deterioration. For perceived stress, 65% (794/1221) improved reliably with 1.8% (22/1221) deterioration. Wilson 95% CIs for all proportions are shown in Tables 4-7.

### Therapist Clustering

The 1221 courses in the paired-assessment sample were delivered by 91 therapists, with a median caseload of 10 (IQR 4-19; range 1-62) courses. In total, 36 (2.9%) courses involved more than 1 therapist across held sessions; the modal therapist was used for clustering. Therapist-level ICCs were small across measures: 0.015 for PHQ-9, 0.029 for GAD-7, and 0.000 for PSS-10, the latter a boundary estimate indicating negligible between-therapist variance. Mean-change estimates and 95% CIs were nearly identical across the therapist-clustered mixed model, cluster-robust SEs, and the unclustered paired *t* test (PHQ-9=6.50, 95% CIs 6.16-6.84, 6.15-6.80, and 6.18-6.76, respectively; GAD-7=7.03, 95% CIs 6.66-7.39, 6.68-7.39, 6.70-7.35, respectively; and PSS-10=8.20; 95% CIs 7.82-8.58, 7.77-8.63, 7.82-8.58, respectively), indicating that accounting for therapist clustering did not alter inference (Table S8 in Multimedia Appendix 3).

### Sensitivity and Robustness Analyses

Effect sizes were stable across matching tiers: Tier 1 only (n=1202, Cohen *d*=1.247, 1.488, and 1.460 for PHQ-9, GAD-7, and PSS-10, respectively), Tier 1+2 (n=1210, Cohen *d*=1.248, 1.487, and 1.460 for PHQ-9, GAD-7, and PSS-10, respectively), and Tier 1+2+3 (n=1221, Cohen *d*=1.247, 1.488, and 1.462 for PHQ-9, GAD-7, and PSS-10, respectively). The maximum difference across tiers was 0.002, indicating that the matching methodology did not meaningfully influence results.

IPW produced effect sizes materially unchanged from the primary analysis. Under the primary weight model (age, gender, baseline symptom scores, and referral pathway), weighted effect sizes were Cohen *d*=1.27 for PHQ-9, Cohen *d*=1.50 for GAD-7, and Cohen *d*=1.48 for PSS-10 (unweighted: 1.25, 1.49, and 1.46), with weighted mean changes of 6.52, 7.09, and 8.25 points, and an effective sample size of approximately 1118 after weight stabilization and trimming. The secondary weight model, additionally including the number of sessions and treatment duration, yielded materially identical results (Cohen *d*=1.27, 1.50, and 1.48). These analyses address attrition among baseline-screened clients only; attrition at the no-screening stage (n=1715) cannot be modeled (Table S7 in Multimedia Appendix 3).

Under the zero-change analysis, in which every baseline-screened client without an end-of-treatment assessment was assigned a change of zero, pooled effect sizes were Cohen *d*=0.41 (PHQ-9), Cohen *d*=0.50 (GAD-7), and Cohen *d*=0.48 (PSS-10). This scenario assumes no symptom change in any nonassessed client and is reported as a reference scenario rather than an estimate.

Restricting the paired-assessment sample to courses whose end-of-treatment screening was completed within 60 days of the last-held session (n=1166; 55 courses excluded) left effect sizes materially unchanged: Cohen *d*=1.25 (PHQ-9), 1.49 (GAD-7), and 1.46 (PSS-10), with a maximum absolute difference of 0.005 from the primary estimates (Table S11 in Multimedia Appendix 3).

### Exploratory Moderator Analyses

In exploratory moderator models (complete case n=1142; Table S9 in Multimedia Appendix 3), higher baseline severity was the dominant correlate of greater symptom change on all 3 measures (3.2-3.4 points of additional change per SD of baseline score), consistent with greater room for improvement and regression to the mean. The number of held sessions was negatively associated with symptom change on all measures (−0.46 to −0.88 points per SD of held sessions), conditional on baseline severity and the other covariates. One possible explanation is confounding by indication. Clients who improve more slowly or present with more persistent difficulties may accrue more sessions. This cannot be established from the present data, in which session count is not randomly assigned but determined jointly by client and therapist during care. The association should not be interpreted as a dose-response relationship in either direction. Remaining covariates showed small or inconsistent associations. These analyses are descriptive, unadjusted for multiplicity, and not causally interpretable.

### Patient-Reported Satisfaction

Patient-reported satisfaction with care was high across all evaluated domains (Table 8). Mean satisfaction with the psychologist was 8.96 (SD 1.45; n=1206), with communication and service 8.84 (SD 1.49; n=1173), and with the platform 8.55 (SD 1.71; n=1211). Recommendation likelihood was also high, with a mean score of 8.61 (SD 1.88, n=1197).

**Table 8:** Client satisfaction and recommendation likelihood ratings.

Domain^a^	Participants, n	Score, mean (SD)	Score, median (IQR)
Psychologist satisfaction	1206	8.96 (1.45)	10.0 (8.0–10.0)
Communication satisfaction	1173	8.84 (1.49)	9.0 (8.0–10.0)
Platform satisfaction	1211	8.55 (1.71)	9.0 (8.0–10.0)
Recommendation likelihood	1197	8.61 (1.88)	9.0 (8.0–10.0)

^a^Satisfaction items rated on a 1-10 Likert scale (1=not at all satisfied, 10=completely satisfied). Recommendation likelihood rated on the same scale. Collected at end-of-treatment screening.

## Discussion

### Principal Findings

This retrospective service evaluation aimed to describe symptom change and patient satisfaction associated with routine online psychological therapy delivered within an insurance-based care setting; both aims were addressed in a paired-assessment sample of 1221 clients. Within the paired-assessment sample, substantial reductions were observed in symptoms of depression, anxiety, and perceived stress from baseline to the end-of-treatment assessment. Large within-sample effect sizes were observed across outcome measures, and the majority of clients met the ≥50% reduction criterion on depression and anxiety, with a smaller proportion meeting it on perceived stress. These are observed changes within a self-selected paired-assessment sample and are reported as associations, not as estimates of a treatment effect.

In addition, patient-reported satisfaction with care was consistently high among clients who returned the end-of-treatment assessment. This is consistent with acceptability of the service model to those clients; it does not establish feasibility, which was not formally evaluated.

In reliable change analysis using the Jacobson-Truax criterion [28], 56% (688/1221)-65% (794/1221) of clients showed improvement exceeding measurement error, which is a measurement-precision criterion rather than evidence of clinical significance. Deterioration rates remained below 2% for all measures; in the absence of a control group, these rates describe observed outcomes and cannot be attributed to the intervention.

The pattern of results was stable across the prespecified and review-prompted robustness analyses. Effect sizes varied by less than 0.01 across screening-to-course matching tiers and by at most 0.005 when end-of-treatment assessments were restricted to a 60-day window after the last session. Accounting for therapist clustering left all CIs materially unchanged, and IPW for attrition among baseline-screened clients yielded effect sizes of 1.27-1.50. Under the assumption that every baseline-screened client without an end assessment experienced no change, effect sizes were 0.41-0.50. These analyses bound specific threats but do not resolve the central one. Paired assessments were available for 1221 of the 5318 (23%) unique clients with at least 1 held session, and the 1715 clients without a baseline screening cannot be represented in any weighted analysis; outcome-related missingness therefore cannot be excluded. The gap between the weighted and zero-change estimates (1.27-1.50 vs 0.41-0.50) shows how sensitive the magnitude is to the missingness assumption, and the primary effect sizes of 1.25-1.49 should be read as describing the paired-assessment sample under a favorable assumption rather than as an estimate for the service population.

A key strength of this evaluation is the use of routinely collected outcome and satisfaction data from a large, real-world clinical population, enhancing ecological validity and relevance for service development. The inclusion of multiple standardized outcome measures also allows comparison with previous real-world evaluations using similar instruments.

### Comparison With Previous Work

Clients in the present sample completed an average of 4.7 therapy sessions, lower than reported in previous real-world evaluations of online and blended care, for example, study by Lungu et al [6] (average of 5.2, SD 2.9 sessions) and Owusu et al [7] (average of 6.00, SD 3.55 sessions). In this study, depressive symptoms decreased by a mean of 6.47 points on the PHQ-9 and anxiety symptoms by 7.03 points on the GAD-7, both corresponding to large effect sizes. These reductions are broadly comparable with those reported in previous real-world evaluations. For reference, Lungu et al [6] reported mean reductions of 5.20 points on the PHQ-9 and 5.65 points on the GAD-7, while Owusu et al [7] reported mean reductions of 8.01 points on the PHQ-9 and 7.02 points on the GAD-7, in both cases among clients with elevated baseline symptoms. Comparable PSS-10 data were not reported in these 2 reference evaluations, which focused on depression and anxiety. The present finding of a large within-sample effect on perceived stress (Cohen *d*=1.46) therefore adds to the limited real-world evidence base for video-based therapy on stress outcomes specifically. The mean of 4.7 (SD 1.67) sessions describes the observed distribution in the paired-assessment sample and should not be read as a recommended treatment dose; course length and clinical trajectory are mutually determined during care.

Outcome patterns also differed across symptom domains in ways consistent with previous literature. While improvements in depression and anxiety were pronounced, reductions in perceived stress were more modest. One possible explanation is that the PSS-10 captures the extent to which individuals perceive their lives as unpredictable, uncontrollable, and overloaded [18,34]; such pressures may be less directly modifiable within a brief course of psychological therapy.

The magnitude of observed change is consistent with contemporaneous at-scale telehealth cohorts. In a large routine-care evaluation of therapist-delivered video psychotherapy (n=2984), Forand et al [10] reported reliable improvement in 65.8% (95% CI 64%-67.5%) of clients and within-sample effect sizes above 0.9 on the PHQ-9 and GAD-7; a workforce mental health platform evaluation (n=52,929) reported effect sizes of 1.61 and 1.82 among clients with elevated baseline symptoms [11]. The present effect sizes (1.25-1.49) fall within the upper range of, and are consistent with, these completer-based paired designs (a design feature shared by all 3 evaluations, as is provider-run authorship). More broadly, recent comparative evidence indicates that remotely delivered therapy achieves outcomes comparable with in-person care. A 2024 meta-analysis of 54 randomized trials found no meaningful difference between therapist-guided remote and in-person CBT (standardized mean difference −0.02, 95% CI −0.12 to 0.07) [8], routine National Health Service data showed equivalent outcomes for remote and face-to-face delivery [9], and video- and telephone-delivered sessions have shown no outcome difference in routine care [35].

The small therapist-level ICCs observed here (0.000-0.029) are consistent with reports from remote and low-intensity routine care: therapist ICCs of 0%-1.3% in routine low-intensity services monitored with the PHQ-9 and GAD-7 [36] and of at most 0.02 in video-delivered psychotherapy [37]. They fall below the 5% weighted average reported across naturalistic psychotherapy settings [38,39]. Several features of the service model may be relevant to this pattern, including uniform therapist qualification requirements and structured onboarding, although this evaluation cannot identify the mechanism. No published therapist-ICC benchmark exists for the PSS-10.

We did not identify a peer-reviewed study published between 2020 and 2026 reporting isolable symptom outcomes for therapist-delivered synchronous video psychotherapy in Nordic or German-speaking routine or insurance-based care, as distinct from randomized or feasibility trials; the present evaluation adds to this limited evidence base. The nearest evidence is a multicenter randomized trial of integrated mental health specialist video consultations in German primary care, which found small effects relative to usual care [40].

### Implications for Service Development

The evaluation surfaces 5 design lessons for implementers, each tied to a limitation in these data. First, baseline screening should be mandatory before the first session in all referral pathways. Coverage was substantially higher in the pathway with mandatory presession screening than where screening was merely encouraged at registration, so pathway design explains part of the observed attrition structure. Second, closure reasons should be captured in structured form; the current binary status field leaves the reasons behind noncompletion unanalyzable at scale. Third, end-of-treatment assessment should be integrated into the final session rather than dispatched after closure; returned screenings arrived a median of 1.2 days after the last session, so the gap is in return rates, not timing. Fourth, therapist-level outcome variation can be monitored routinely using ICCs as a quality-assurance signal; the values observed here provide a baseline. Fifth, validated satisfaction instruments would strengthen the interpretability of patient-reported satisfaction relative to the current single-item measures.

### Limitations

Several limitations should be acknowledged. First, this study was conducted as a retrospective observational service evaluation without a comparison group. Consequently, causal conclusions cannot be drawn, and the observed symptom improvements may partly reflect regression to the mean, or spontaneous symptom improvement over time. Outcomes were assessed only up to the end-of-treatment assessment, so the durability of change beyond the end of therapy is unknown.

Second, analyses were restricted to the paired-assessment sample, and a missing-not-at-random mechanism cannot be excluded. Completion of the end-of-treatment screening may be related to satisfaction or improvement. In total, 3 observations bound this concern without eliminating it. Baseline symptom severity did not differ significantly between baseline-only clients and the paired-assessment sample (all *P*≥.48). The inverse-probability-weighted analysis, which assumes missingness at random given covariates, and the zero-change analysis, which assumes no change in any nonassessed client, provide contrasting reference points. The zero-change scenario is not a worst case; nonassessed clients could in principle have deteriorated, in which case the true population change would fall below the zero-change value. Therapist-recorded closure status indicates that 85%-87% of clients without paired assessments were judged by their therapist to have completed treatment successfully within the course framework. This is weak supporting evidence and does not show that missing outcome data are benign. The judgment is binary, made by the treating therapist without operational criteria, blinding, or independent verification, and therapists have a plausible incentive to record their own courses as successful. It is also formed from the same clinical impression that may shape whether an end-of-treatment screening is returned, and is therefore not independent of the missingness mechanism it is invoked to bound. It is consistent with a measurement gap rather than treatment failure, but does not demonstrate one.

Third, inclusion in the paired-assessment sample is defined by assessment return, not by clinically agreed treatment completion, and the two should not be equated. The structured closure field distinguishes successful from unsuccessful completion but not the reasons behind unsuccessful closure. Prospective structured capture of closure reasons, as outlined in the Implications for Service Development section, would resolve this ambiguity.

Fourth, no structured diagnostic information was available. Presenting-concern categories derive from referral records or the pretreatment assessment consultation; they are not diagnoses, they are recorded in 2 pathway-specific taxonomies that were not harmonized, and no structured category exists for a small direct-booking subgroup (4.2% of courses). Together with missing age and gender data for a subset of clients, this limited the granularity of subgroup analyses. This heterogeneity constrains interpretation in 3 ways. First, the reported effect sizes average across clients with materially different presentations. In both pathways, the most frequently recorded concerns were stress-, adjustment-, or life circumstance–related rather than a defined mental disorder (the 2 largest categories alone accounted for 256/706, 36.3% and 248/377, 65.7% of recorded concerns, respectively). Natural symptom trajectories may differ across these groups, and the present data do not allow us to determine how much of the observed pre-post change reflects case-mix rather than the therapy delivered. Second, because outcome-relevant subgroups cannot be defined reliably from nondiagnostic labels recorded in 2 unharmonized taxonomies, we could not test whether symptom change differed across presenting problems. Third, differences between the present estimates and those from diagnostically defined samples may reflect case-mix rather than the intervention or its delivery, which limits benchmarking against studies recruiting on diagnostic criteria.

Fifth, intervention fidelity was not directly assessed. Adherence to CBT principles was not measured with any adherence or competence rating scale, no sessions were recorded or rated, and no treatment manual or session-content log was in use. Individual clinical supervision was not mandated at the service level during the study period, so therapist performance was monitored through biannual documentation audits rather than direct supervisory oversight. What is documented is structural; Danish authorization, documented before CBT experience, and a structured onboarding protocol. The extent to which the therapy delivered corresponded to CBT is therefore unknown, and the observed outcomes cannot be attributed to CBT as a specified treatment.

Sixth, the satisfaction items are single-item service-evaluation measures. Single-item measures can show usable validity but preclude internal-reliability estimation [41], and satisfaction ratings of this kind are susceptible to ceiling effects—medians here were 9-10 on a 10-point scale—as well as acquiescence and social-desirability bias; framing alone has been shown experimentally to shift favorable response rates by several percentage points [42]. Ratings were, moreover, available predominantly for clients who completed the end-of-treatment screening.

Seventh, no validated clinical cutoff or generalizable minimal important change is established for the PSS-10; available Danish and German norm-based cut points are distributional rather than clinically anchored [43,44]. The only anchor-based estimate of clinically important change, from the Danish validation sample (n=64, unreplicated), is 11 points or a 28% relative reduction (Eskildsen [29]); the mean change observed here (8.2 points, 33.8%) exceeds the relative but not the absolute estimate. Perceived stress outcomes are therefore reported continuously and as reliable change; neither the ≥50% reduction criterion nor the RCI should be read as establishing clinical significance for this measure.

Eighth, the service operates within the Danish employer- and insurance-based coverage model, in which access, referral incentives, and session-count expectations are shaped by insurance products. Generalizability to publicly funded systems, self-pay markets, or other regulatory contexts is unknown.

Finally, outcome measures were self-reported and may be influenced by reporting bias, expectancy effects, or social desirability. All authors are salaried employees of we.care, and no independent statistician reviewed the analysis. Mitigation measures—prespecification of the primary analysis set, transparent labeling of post hoc decisions, and public deposit of aggregate data, analysis code, and a codebook at the OSF—are described in the Methods and Conflicts of Interest sections; residual interpretive bias cannot be fully excluded, and the findings should be read as a transparent account of outcomes in 1 service rather than as evidence of efficacy.

### Conclusions

This service evaluation describes pre-post symptom change, the proportions of clients meeting a descriptive ≥50% reduction criterion and showing reliable improvement, and patient-reported satisfaction in the paired-assessment sample of a routine online psychological therapy service delivered within an insurance-based care setting. Observed within-sample effect sizes were large and broadly consistent with those reported in previous real-world evaluations of online and blended care [6,7]. They describe a sample comprising 23% of clients with at least 1 held session and are not necessarily representative of the full service population. Because the evaluation used an uncontrolled pre-post design restricted to clients with paired assessments, these changes cannot be causally attributed to the therapy delivered; findings should be interpreted as a description of outcomes under routine care conditions. The results are consistent with—but do not themselves demonstrate—the potential utility of therapist-delivered online care models as a component of routine mental health service delivery. Randomized or otherwise adequately controlled studies, with longer follow-up, are required to establish comparative effectiveness and durability of gains. For service implementers, the more immediate lessons are procedural; mandatory presession baseline screening, structured closure-reason capture, and end-of-treatment assessment integrated into the final session would materially strengthen both routine monitoring and future evaluation.

## Appendix

Multimedia Appendix 1 STROBE checklist.

Multimedia Appendix 2 Screening-to-course linkage diagram. Of 8352 screenings submitted during the study period, 5964 were matched to a therapy course via a 3-tier deterministic strategy: tier 1, exact 8-digit client identifier (n=5887); tier 2, unambiguous Levenshtein-distance-1 fuzzy match (n=27); tier 3, manual review following hand-verification of 79 candidate IDs (n=50). A total of 2388 screenings remained unmatched (no confident course link). Where a client had multiple courses, screenings were assigned to the nearest course using Voronoi date-based nearest-assignment (soft-capped at 180 days). Screenings falling outside the per-pathway temporal assessment window or violating the temporal-coherence criterion were excluded before analysis (refer to Methods section).

Multimedia Appendix 3 Extended aggregate outputs.

## Abbreviations

CBT: cognitive behavioral therapy
DORA: Digital Operational Resilience Act
GAD-7: 7-item Generalized Anxiety Disorder
GDPR: General Data Protection Regulation
ICC: intraclass correlation coefficient
IPW: inverse-probability weighting
PHQ-9: 9-item Patient Health Questionnaire
PSS-10: 10-item Perceived Stress Scale
RCI: reliable change index
STROBE: Strengthening the Reporting of Observational Studies in Epidemiology

## Data Availability

The aggregate data and analysis outputs supporting this evaluation are openly available in the Open Science Framework repository [33] under a CC-BY 4.0 license. The deposit contains the aggregate values underlying Figures 1 and 2 and Tables 1-8, the presenting-concern distributions, the session-attendance metrics, the screening-to-course matching-tier comparison, the inverse-probability-weighted and zero-change attrition analyses, the therapist-level variance components and intraclass correlations, and the exploratory moderator model, together with a README, a codebook documenting every file and column, and the Python analysis code with pinned dependencies. From these materials, every descriptive statistic, effect size, confidence interval and derived proportion reported in Tables 1-8 can be recomputed from the deposited values; the participant flow and the score distributions in Figures 1 and 2 can be reproduced; the weighted and zero-change effect sizes can be checked against the primary estimates; and the full analytic pipeline, including the code that generates the Multimedia Appendix tables, can be inspected. The individual-level analytical dataset is not deposited, as it constitutes personal data under the EU General Data Protection Regulation and Danish data-protection law; the deposited files contain no individual-level rows, and categories with fewer than five clients are combined. Analyses that take that dataset as input therefore cannot be re-executed from the deposit: the end-of-treatment assessment-window sensitivity analysis, the closure-status distribution by attrition cohort, the confidence intervals for the three therapist-clustering estimation approaches, and the record linkage, whose extraction scripts require database and interface credentials. Requests for access to de-identified individual-level data for specified academic research purposes may be directed to the corresponding author and will be considered subject to formal data-transfer agreement and applicable institutional and legal clearance.
