# Supplementary material for: Routine Online Psychological Therapy in an Insurance-Based Care Setting: Retrospective Service Evaluation of Real-World Outcomes
Source: JMIR Form Res. 2026 Sep 25;10:e101074. doi: 10.2196/101074 (PMC13653202; doi:10.2196/101074)
Supplement: Supplementary file 1 [file formative_v10i1e101074_app1.docx]

**STROBE Statement — checklist of items to be reported**

*Manuscript: Routine Online Psychological Therapy in an Insurance-Based Care Setting: Retrospective Service Evaluation of Real-World Outcomes (JMIR Formative Research, #101074)*

Retrospective service evaluation of routinely collected health data (cohort / cross-sectional items).

Reported per STROBE (von Elm et al., 2007); the study uses routinely collected health data, for which RECORD (Benchimol et al., 2015) extends items 1, 6, 7, 12, 13, 19, and 22 — cited in Methods.

The “Reported in section/page” column cites manuscript SECTION names rather than page numbers, which are unstable across revision and typesetting; no page numbers are asserted.

| **Item #** | **Recommendation** | **Reported in section/page** |
| --- | --- | --- |
| 1 | Title and abstract — (a) study design in title/abstract; (b) balanced summary in abstract. | Title; Abstract |
| 2 | Background/rationale — scientific background and rationale. | Introduction |
| 3 | Objectives — specific objectives/hypotheses. | Introduction (final paragraph) |
| 4 | Study design — key design elements early in the paper. | Methods — Study Design |
| 5 | Setting — setting, locations, relevant dates (recruitment, exposure, follow-up, data collection). | Methods — Setting and Service Description |
| 6 | Participants — (a) eligibility criteria, sources, selection methods; (b) [cohort] matching criteria / N exposed-unexposed (n/a here — state). | Methods — Participants; Data Linkage. (b) Not applicable — uncontrolled single-cohort design, no matching |
| 7 | Variables — outcomes, exposures, predictors, confounders, effect modifiers; diagnostic criteria. | Methods — Measures (Clinical Outcome Measures; Presenting Concerns; Patient-Reported Satisfaction; Demographic and Treatment Variables) |
| 8 | Data sources/measurement — for each variable, sources and assessment methods; comparability across groups. | Methods — Measures (Clinical Outcome Measures); Data Linkage |
| 9 | Bias — efforts to address potential sources of bias. | Methods — Statistical Analyses (attrition sensitivity analyses); Discussion — Limitations |
| 10 | Study size — how study size was arrived at. | Methods — Participants; Results — Study Population and Treatment Characteristics |
| 11 | Quantitative variables — handling of quantitative variables/groupings. | Methods — Clinical Outcome Measures (cutoffs, reliable change criteria); Statistical Analyses |
| 12 | Statistical methods — (a) all methods incl. confounding control; (b) subgroups/interactions; (c) missing data; (d) loss-to-follow-up/attrition handling; (e) sensitivity analyses. | Methods — Statistical Analyses |
| 13 | Participants — (a) numbers at each stage; (b) reasons for non-participation; (c) flow diagram. | Results — Study Population and Treatment Characteristics; Multimedia Appendix Figure S1 (linkage/flow) |
| 14 | Descriptive data — (a) participant characteristics; (b) missing data per variable; (c) follow-up time. | Results — Study Population and Treatment Characteristics (Table 1); attrition paragraph |
| 15 | Outcome data — numbers of outcome events or summary measures. | Results — Changes in Depression, Anxiety, and Perceived Stress (Tables 2–3) |
| 16 | Main results — (a) unadjusted and adjusted estimates + precision (95% CIs); (b) category boundaries; (c) absolute risk if relevant. | Results — Changes in Depression, Anxiety, and Perceived Stress; Symptom Reduction and Below-Cutoff Status (Tables 3–4) |
| 17 | Other analyses — subgroups, interactions, sensitivity analyses. | Results — Therapist Clustering; Sensitivity and Robustness Analyses; Exploratory Moderator Analyses (Multimedia Appendix Tables A7–A12) |
| 18 | Key results — summarise with reference to objectives. | Discussion — Principal Findings |
| 19 | Limitations — sources of bias/imprecision, direction/magnitude. | Discussion — Limitations |
| 20 | Interpretation — cautious overall interpretation. | Discussion — Principal Findings; Comparison With Prior Work; Conclusions |
| 21 | Generalisability — external validity. | Discussion — Limitations; Implications for Service Development |
| 22 | Funding — role of funders; original study the data derive from. | Funding |
