# Supplementary material for: Routine Online Psychological Therapy in an Insurance-Based Care Setting: Retrospective Service Evaluation of Real-World Outcomes
Source: JMIR Form Res. 2026 Sep 25;10:e101074. doi: 10.2196/101074 (PMC13653202; doi:10.2196/101074)
Supplement: Supplementary file 2 [file formative_v10i1e101074_app3.docx]

**Appendix S1: Extended aggregate outputs**

This appendix supplements Tables 1-8 of the main manuscript with additional aggregate statistics: detailed score distributions, matching-tier sensitivity, full Reliable Change Index counts, baseline-severity subgroup analyses, presenting concerns (referral- and assessment-derived categories), and per-item satisfaction detail (S1–S6 and S5b), together with the R1 revision additions — attrition sensitivity analyses, therapist clustering, an exploratory moderator model, change-score effect sizes, end-window sensitivity, and closure status (S7–S12). No individual-level data are reported; cells with n < 5 are suppressed and all values are aggregate.

Table S1. Per-measure score distributions at baseline and treatment completion.

| **Measure** | **Timepoint** | **N** | **M (SD)** | **Min** | **P5** | **P10** | **P25** | **Median** | **P75** | **P90** | **P95** | **Max** |
| --- | --- | --- | --- | --- | --- | --- | --- | --- | --- | --- | --- | --- |
| PHQ-9 | Baseline | 1221 | 12.47 (5.19) | 0.0 | 4.0 | 6.0 | 8.0 | 13.0 | 16.0 | 20.0 | 21.0 | 27.0 |
|  | End of treatment | 1221 | 6.0 (4.64) | 0.0 | 0.0 | 1.0 | 3.0 | 5.0 | 8.0 | 13.0 | 16.0 | 24.0 |
| GAD-7 | Baseline | 1221 | 12.21 (4.73) | 1.0 | 4.0 | 5.0 | 9.0 | 13.0 | 16.0 | 18.0 | 19.0 | 21.0 |
|  | End of treatment | 1221 | 5.18 (4.12) | 0.0 | 0.0 | 1.0 | 2.0 | 4.0 | 7.0 | 12.0 | 14.0 | 21.0 |
| PSS-10 | Baseline | 1221 | 23.18 (5.61) | 4.0 | 13.0 | 15.0 | 20.0 | 24.0 | 27.0 | 30.0 | 32.0 | 39.0 |
|  | End of treatment | 1221 | 14.98 (6.53) | 0.0 | 5.0 | 7.0 | 10.0 | 14.0 | 19.0 | 24.0 | 27.0 | 39.0 |

*Percentiles (P5–P95) describe the distribution of raw scores within the analytical sample.*

Table S2. Within-sample effect sizes (Cohen's d) stratified by matching tier.

| **Matching tier** | **N** | **d (PHQ-9) [95% CI]** | **d (GAD-7) [95% CI]** | **d (PSS-10) [95% CI]** |
| --- | --- | --- | --- | --- |
| Tier 1 only (exact) | 1202 | 1.247 [1.172, 1.322] | 1.488 [1.406, 1.571] | 1.46 [1.378, 1.541] |
| Tier 1 + 2 (exact + Levenshtein-1) | 1210 | 1.248 [1.173, 1.323] | 1.487 [1.405, 1.569] | 1.46 [1.379, 1.541] |
| Tier 1 + 2 + 3 (all, including manual) | 1221 | 1.247 [1.172, 1.322] | 1.488 [1.406, 1.569] | 1.462 [1.381, 1.542] |

*Cohen's d = mean(change) / SD(baseline); 95% CI via non-central t-distribution.*

Table S3. Reliable Change Index classification counts by measure.

| **Measure** | **N analysed** | **Reliably improved, n (%)** | **No reliable change, n (%)** | **Reliably deteriorated, n (%)** | **Test-retest r** |
| --- | --- | --- | --- | --- | --- |
| PHQ-9 | 1221 | 688 (56.3%) | 524 (42.9%) | 9 (0.7%) | 0.84 |
| GAD-7 | 1221 | 747 (61.2%) | 461 (37.8%) | 13 (1.1%) | 0.83 |
| PSS-10 | 1221 | 794 (65.0%) | 405 (33.2%) | 22 (1.8%) | 0.87 |

*RCI = (pre − post) / SE_diff, where SE_diff = √(2) × SD_baseline × √(1 − r). Reliably improved if RCI > 1.96; deteriorated if RCI < −1.96.*

Table S4. Pre-post change and effect size by baseline severity stratum.

| **Measure** | **Stratum** | **N** | **Pre M (SD)** | **Post M (SD)** | **Δ M** | **d [95% CI]** |
| --- | --- | --- | --- | --- | --- | --- |
| PHQ-9 | Below cutoff (<10) | 381 | 6.43 (2.14) | 3.65 (2.96) | 2.77 | 1.298 [1.162, 1.435] |
|  | At or above cutoff (10-19) | 710 | 14.11 (2.65) | 6.6 (4.55) | 7.5 | 2.836 [2.671, 3.001] |
|  | Severe (>=20) | 130 | 21.28 (1.43) | 9.58 (5.73) | 11.69 | 8.204 [7.182, 9.225] |
| GAD-7 | Below cutoff (<8) | 218 | 4.93 (1.62) | 2.9 (2.28) | 2.02 | 1.252 [1.073, 1.43] |
|  | At or above cutoff (8-14) | 564 | 11.17 (1.97) | 4.99 (3.63) | 6.18 | 3.141 [2.94, 3.343] |
|  | Severe (>=15) | 439 | 17.17 (1.87) | 6.56 (4.83) | 10.62 | 5.671 [5.283, 6.058] |
| PSS-10 | Below cutoff (<14) | 69 | 10.62 (2.49) | 9.3 (5.31) | 1.32 | 0.531 [0.274, 0.787] |
|  | Moderate (14-26) | 807 | 21.53 (3.3) | 14.47 (5.97) | 7.06 | 2.141 [2.016, 2.266] |
|  | High (>=27) | 345 | 29.56 (2.47) | 17.31 (7.05) | 12.24 | 4.959 [4.573, 5.345] |

*Thresholds: PHQ-9 <10 / 10–19 / ≥20; GAD-7 <8 / 8–14 / ≥15; PSS-10 <14 / 14–26 / ≥27. Strata with n < 5 suppressed.*

Table S5. Presenting concerns at treatment initiation (referral-derived presenting-concern categories, encouraged-screening pathway).

Coverage: 706 of 791 encouraged-screening-pathway courses (89.3%).

| **Presenting concern** | **n** | **% of tagged** |
| --- | --- | --- |
| Stress (including work-related stress) | 160 | 22.7% |
| Relative of someone who is seriously ill | 96 | 13.6% |
| Anxiety symptoms | 72 | 10.2% |
| Depression | 69 | 9.8% |
| Anxiety | 68 | 9.6% |
| Depressive symptoms | 66 | 9.3% |
| Adjustment disorder | 57 | 8.1% |
| Acute stress reaction | 47 | 6.7% |
| People affected by serious and/or disabling illness | 32 | 4.5% |
| Termination / Dismissal from work | 8 | 1.1% |
| Well-being / Poor well-being | 6 | 0.8% |
| Abortion | 5 | 0.7% |
| Other (combined, each n<5) | 20 | 2.8% |

*Referral-derived presenting-concern categories from a fixed 28-category taxonomy (recorded once per course; one category per course; not diagnoses). The category reflects the reason for referral recorded by the referring organisation’s health professionals, not the treating psychologist. Percentages are of encouraged-screening-pathway courses with a recorded category. Courses on the mandatory-screening pathway carry no such categories. The n<5 tail is combined into a single “Other” row.*

Table S5b. Presenting concerns at treatment initiation (assessment-derived categories, mandatory-screening pathway).

Coverage: 377 of 379 mandatory-screening-pathway courses (99.5%). A direct-booking subgroup (n=51) bypasses the assessment consultation by design and is not applicable. Whole-sample structured coverage across both pathways: 88.7%.

| **Presenting concern** | **n** | **% of assessed** |
| --- | --- | --- |
| Psychological stress | 131 | 34.7% |
| Poor well-being and stress reaction | 117 | 31.0% |
| Relationship difficulties | 31 | 8.2% |
| Relative of a person with mental or physical illness | 26 | 6.9% |
| Anxiety | 26 | 6.9% |
| Depression | 21 | 5.6% |
| Death in the family | 12 | 3.2% |
| Serious illness | 8 | 2.1% |
| Other (combined, each n<5) | 5 | 1.3% |

*Assessment-derived presenting-concern categories from a fixed 12-category taxonomy (recorded once per course by the assessing psychologist at the initial assessment consultation; one category per course; not diagnoses). Percentages are of mandatory-screening-pathway courses with a recorded category. A direct-booking subgroup that bypasses the assessment consultation carries no such category and is excluded from the denominator. The n<5 tail is combined into a single “Other” row.*

Table S6. Per-item satisfaction and recommendation likelihood breakdown.

| **Item** | **N** | **M (SD)** | **Median [Q1–Q3]** | **% rating 9 or 10** | **% rating 1–4** |
| --- | --- | --- | --- | --- | --- |
| Psychologist satisfaction | 1206 | 8.96 (1.45) | 10.0 [8.0–10.0] | 69.8% | 1.4% |
| Communication satisfaction | 1173 | 8.84 (1.49) | 9.0 [8.0–10.0] | 65.6% | 2.1% |
| Platform satisfaction | 1211 | 8.55 (1.71) | 9.0 [8.0–10.0] | 58.5% | 2.8% |
| Recommendation likelihood | 1197 | 8.61 (1.88) | 9.0 [8.0–10.0] | 63.3% | 3.9% |

*All items rated on a 1–10 Likert scale; collected at end-of-treatment screening.*

Table S7. Attrition sensitivity analyses: inverse-probability-of-completion weighting and a zero-change floor.

| **Measure** | **Unweighted d** | **IPW primary: Δ (weighted)** | **IPW primary: d [dz]** | **IPW secondary: Δ (weighted)** | **IPW secondary: d [dz]** | **Zero-change floor d** |
| --- | --- | --- | --- | --- | --- | --- |
| PHQ-9 | 1.247 | 6.522 | 1.267 [1.247] | 6.533 | 1.266 [1.246] | 0.414 |
| GAD-7 | 1.488 | 7.088 | 1.502 [1.377] | 7.111 | 1.497 [1.385] | 0.495 |
| PSS-10 | 1.462 | 8.251 | 1.481 [1.206] | 8.294 | 1.475 [1.213] | 0.478 |

*IPW = inverse-probability-of-completion weighting; weights recover the mean change had all baseline-screened clients completed, under missing-at-random given baseline covariates. Primary weight model = baseline covariates only; secondary = + held sessions and duration. Effective sample size (post-trim): primary 1118.5, secondary 1084.1. Δ = weighted mean change; d / dz = weighted effect sizes (baseline-SD and change-score standardisers). The no-screening cohort (n=1,715) has no baseline scores and is unmodellable. The zero-change floor assumes every baseline-screened non-completer had zero change — a lower bound, not an estimate.*

Table S8. Therapist clustering: caseload, intraclass correlation, variance components, and mean-change intercept.

| **Measure** | **Therapists (N)** | **Caseload median [IQR]** | **Mean-change intercept** | **Mixed-model 95% CI** | **Cluster-robust 95% CI** | **Paired-t 95% CI** | **ICC** | **τ² (between)** | **σ² (residual)** |
| --- | --- | --- | --- | --- | --- | --- | --- | --- | --- |
| PHQ-9 | 91 | 10 [4–19] | 6.501 | [6.16, 6.84] | [6.15, 6.80] | [6.18, 6.76] | 0.0154 | 0.4118 | 26.4018 |
| GAD-7 | 91 | 10 [4–19] | 7.026 | [6.66, 7.39] | [6.68, 7.39] | [6.75, 7.32] | 0.0291 | 0.7642 | 25.4811 |
| PSS-10 | 91 | 10 [4–19] | 8.199 | [7.82, 8.58] | [7.77, 8.63] | [7.82, 8.58] | 0.0000 | 0.0000 | 48.1801 |

*Random-intercept mixed models (REML). ICCs are small relative to the ~0.05 psychotherapy benchmark (Baldwin & Imel 2013). For PSS-10, τ² → 0 is a genuine REML boundary estimate (no detectable between-therapist variance); the cluster-robust SE provides inference regardless of the variance component. N therapists = 91; caseload = courses per therapist. Aggregate only — no per-therapist data are reported.*

Table S9. Exploratory moderators of symptom change (standardised coefficients, 95% CI).

| **Predictor** | **PHQ-9 β [95% CI]** | **GAD-7 β [95% CI]** | **PSS-10 β [95% CI]** |
| --- | --- | --- | --- |
| Baseline severity (z) | 3.18 [2.93, 3.43] | 3.43 [3.20, 3.66] | 3.33 [2.96, 3.69] |
| Age (z) | 0.22 [−0.04, 0.47] | 0.37 [0.14, 0.60] | 0.29 [−0.08, 0.65] |
| Gender (Non-female vs Female) | 0.53 [0.01, 1.05] | 0.51 [0.02, 0.99] | 0.62 [−0.14, 1.39] |
| Held sessions (z) | −0.46 [−0.83, −0.09] | −0.57 [−0.92, −0.23] | −0.88 [−1.42, −0.34] |
| Treatment duration (z) | 0.22 [−0.15, 0.59] | 0.28 [−0.06, 0.62] | 0.64 [0.10, 1.17] |
| Referral pathway (mandatory- vs encouraged-screening) | 0.43 [−0.09, 0.95] | 0.26 [−0.23, 0.74] | 0.52 [−0.24, 1.27] |

*MixedLM (REML, therapist random intercept); standardised predictors (z); outcome = symptom change (pre − post). Complete-case n = 1,142 (79 dropped for missing age/gender). The referral-pathway coefficient contrasts the mandatory-screening pathway with the encouraged-screening pathway (reference). EXPLORATORY / descriptive: no multiplicity correction. The negative held-sessions association is most plausibly confounding by indication (clients who improve sooner end sooner), NOT a dose–response effect. Baseline severity is the substantive moderator.*

Table S10. Within-subject change-score effect sizes (Cohen's dz) beside baseline-SD Cohen's d.

| **Measure** | **Baseline-SD d [95% CI]** | **Change-score dz [95% CI]** |
| --- | --- | --- |
| PHQ-9 | 1.247 [1.172, 1.322] | 1.251 [1.176, 1.325] |
| GAD-7 | 1.488 [1.406, 1.569] | 1.374 [1.296, 1.452] |
| PSS-10 | 1.462 [1.381, 1.542] | 1.197 [1.124, 1.271] |

*Cohen's dz = mean(change) / SD(change) — the within-subject (change-score) standardiser, which incorporates the pre–post correlation (Lakens 2013). Baseline-SD d = mean(change) / SD(baseline) is the primary metric for cross-study comparability (Morris & DeShon 2002). Rendered as a standalone table; A2 retains the tier-stratified baseline-SD d.*

Table S11. End-window sensitivity: effect sizes restricted to end screenings within the linkage window.

Sub-sample: end screening submitted ≤ 60 days after the last held session (n = 1,166; 55 courses with a beyond-window end screening excluded).

| **Measure** | **N** | **Mean change** | **Baseline-SD d [95% CI]** | **Change-score dz [95% CI]** | **Δd vs primary** |
| --- | --- | --- | --- | --- | --- |
| PHQ-9 | 1166 | 6.505 | 1.252 [1.176, 1.329] | 1.266 [1.189, 1.343] | +0.005 |
| GAD-7 | 1166 | 7.028 | 1.49 [1.406, 1.573] | 1.376 [1.296, 1.456] | +0.002 |
| PSS-10 | 1166 | 8.163 | 1.458 [1.375, 1.54] | 1.206 [1.13, 1.281] | −0.004 |

*Additive linkage-robustness sensitivity (reviewer point AU7); the primary analysis retains all N = 1,221. End offset = submitted end screening − last held session, from raw timestamps (same anchors as the internal timing diagnostic). Effect sizes use the same estimators and 95% CI framework as the main analysis (Cohen's d on the baseline SD; dz on the change-score SD). Primary baseline-SD d: PHQ-9 1.247, GAD-7 1.488, PSS-10 1.462. Largest absolute shift |Δd| = 0.005 — effect sizes are materially unchanged, so the retained beyond-window end screenings do not drive the results.*

Table S12. Therapist-recorded closure status by assessment-completion cohort.

| **Closure status** | **Paired-assessment sample (n=1,221)** | **Start only (n=2,382)** | **No screening (n=1,713)** |
| --- | --- | --- | --- |
| Successful | 1198 (98.1%) | 2036 (85.5%) | 1487 (86.8%) |
| Unsuccessful | 19 (1.6%) | 302 (12.7%) | 196 (11.4%) |
| Missing (not recorded) | – | 41 (1.7%) | 27 (1.6%) |
| Partially | – | – | – |

*Closure status is therapist-reported at course closure (response to “Er klienten færdigbehandlet indenfor forløbets rammer?”: Yes → successful, No → unsuccessful); “partially” is available in one partner configuration only. Closure coverage is 99.8% of the paired-assessment sample and 98.4% of all courses. The no-screening denominator is 1,713 because 2 erroneously created courses (status “wrong”) were excluded from the closure tallies, whereas the attrition flow reports 1,715 for the same cohort. Closure status is a therapist judgement of whether treatment concluded within the course’s frame, not an independent outcome measure. Cells with n < 5 are suppressed (“–”).*

The screening→course record-linkage flow (matching tiers and manual review) is shown in Supplementary Figure S1 (figS1_linkage).
